# Reconstitution of the recombinant human γ-tubulin ring complex

**DOI:** 10.1098/rsob.200325

**Published:** 2021-02-03

**Authors:** Martin Würtz, Anna Böhler, Annett Neuner, Erik Zupa, Lukas Rohland, Peng Liu, Bram J. A. Vermeulen, Stefan Pfeffer, Sebastian Eustermann, Elmar Schiebel

**Affiliations:** ^1^ Zentrum für Molekulare Biologie der Universität Heidelberg (ZMBH), Im Neuenheimer Feld 282, D-69120 Heidelberg, Germany; ^2^ Centre for Organismal Studies Universität Heidelberg (COS), Im Neuenheimer Feld 230, D-69120 Heidelberg, Germany; ^3^ European Molecular Biology Laboratory (EMBL), Heidelberg Meyerhofstraße 1, 69117 Heidelberg, Germany

**Keywords:** γ-tubulin, microtubules, γ-TuRC

## Abstract

Cryo-electron microscopy recently resolved the structure of the vertebrate γ-tubulin ring complex (γ-TuRC) purified from *Xenopus laevis* egg extract and human cells to near-atomic resolution. These studies clarified the arrangement and stoichiometry of γ-TuRC components and revealed that one molecule of actin and the small protein MZT1 are embedded into the complex. Based on this structural census of γ-TuRC core components, we developed a recombinant expression system for the reconstitution and purification of human γ-TuRC from insect cells. The recombinant γ-TuRC recapitulates the structure of purified native γ-TuRC and has similar functional properties in terms of microtubule nucleation and minus end capping. This recombinant system is a central step towards deciphering the activation mechanisms of the γ-TuRC and the function of individual γ-TuRC core components.

## Graphical abstract

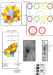

## 1. Introduction

Microtubules are essential cytoskeletal components with multiple important functions in chromosome segregation, cellular transport, cell organization and cell motility. Microtubules are polymers of the heterodimer *α*β-tubulin that dynamically assemble via lateral and longitudinal subunit interactions into a cylindric structure composed of 13 αβ-tubulin protofilaments [1]. Formation of the initial αβ-tubulin oligomers is the rate-limiting step in the *de novo* assembly of microtubules [2]. In cells, complexes containing the tubulin family member γ-tubulin facilitate this step in a controlled manner and thus determine the sites of microtubule formation [3,4].

**Figure 1.**
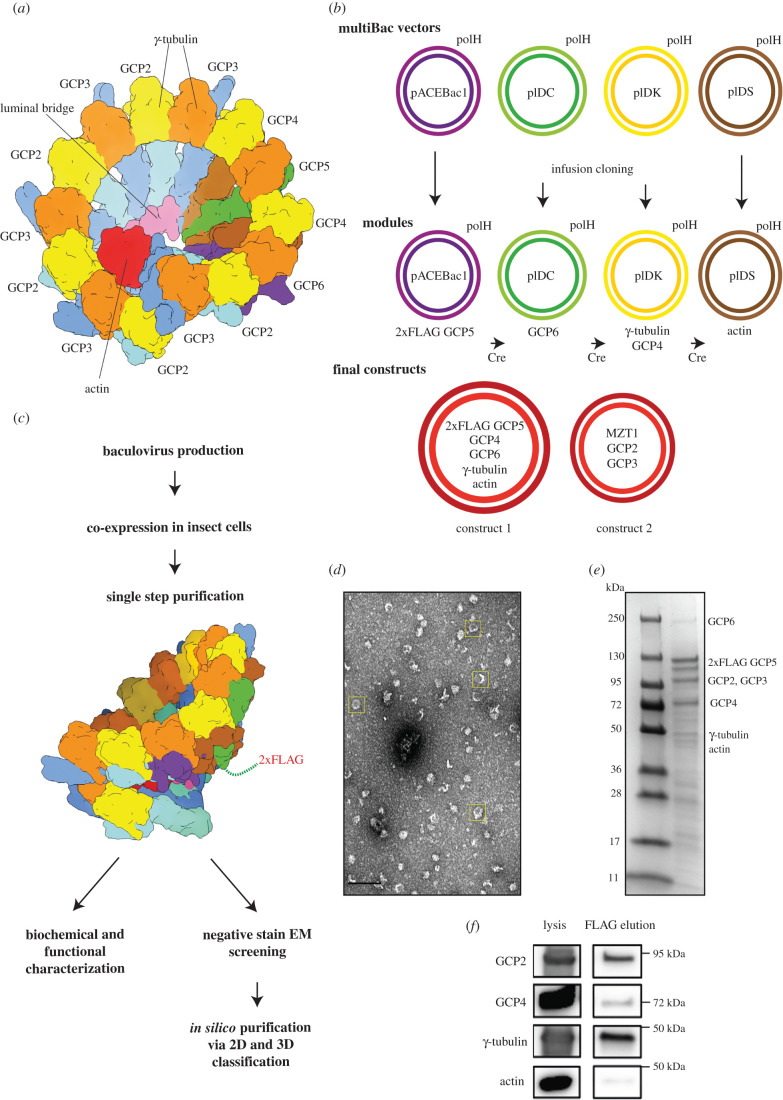
A modularized expression system enables purification of 2xFLAG-tagged recombinant γ-TuRC. (*a*) Schematic representation of the 14-spoke vertebrate γ-TuRC. Colours: GCP2 (light blue), GCP3 (dark blue), γ-tubulin (orange/yellow), GCP4 (brown), GCP5 (green), GCP6 (purple), actin (red) and luminal bridge (pink). (*b*) Cloning strategy for the recombinant γ-TuRC using the MultiBac vectors pACEBac1, PIDC, pIDK and pIDS with polyhedrin (polH) expression cassette. Human genes of the γ-TuRC were inserted via Infusion cloning. The ‘modules', plasmids with one or two genes of interest, were combined via subsequent Cre-recombination. Construct 1 for γ-TuRC expression consists of 2x*FLAG-GCP5*, *GCP6*, *GCP4*, *TUBG1* and *ACTB*. This construct was used for bacmid and virus production and for protein expression. It was complemented with construct 2 coding for *MZT1*, *GCP2* and *GCP3*. (*c*) Recombinant γ-TuRC was isolated via FLAG affinity purification and FLAG peptide elution in a single-step protocol and used for subsequent characterization using negative stain EM as well as biochemical approaches. Scheme of the γ-TuRC indicating the approximate position of the 2xFLAG tag at the N-terminus of GCP5. Colours as in (*a*). (*d*) Section of a negative stain EM micrograph of human recombinant γ-TuRC after FLAG affinity purification. Yellow boxes indicate exemplary γ-TuRC particles. Scale bar: 100 nm. (*e*) Section of Coomassie Blue-stained SDS-PAGE gel of γ-TuRC elution after FLAG affinity purification. (*f*) Cell lysate and FLAG affinity-purified recombinant γ-TuRC (FLAG elution) were probed using immunoblotting against the indicated antibodies. See electronic supplementary material, figure S2 for uncropped images.

In higher eukaryotes, nucleation of microtubules is mediated by the γ-tubulin ring complex (γ-TuRC) [5]. High-resolution structural information on the γ-TuRC was limited to a few isolated components [6,7], until recent advances in cryo-electron microscopy (cryo-EM) facilitated resolving the structure of purified vertebrate γ-TuRCs to near–atomic resolution [8–10]. These analyses showed that the γ-TuRC contains γ-tubulin, five paralogous gamma-ring complex proteins (GCPs, GCP2 to 6), mitotic spindle organizing protein 1 (MZT1), MZT2 and actin in a defined stoichiometry [8–11]. The γ-TuRC comprises 14 asymmetrically arranged spokes (pairs of GCP and γ-tubulin; figure 1*a*) with the order (GCP2-3)_4_-GCP4-GCP5-GCP4-GCP6-(GCP2-3)_1_. The GCP proteins interact with each other via their N-terminal gamma-tubulin ring protein 1 (GRIP1) domains, while their C-terminal GRIP2 domains bind one molecule of γ-tubulin each. A prominent feature of the γ-TuRC is a structural scaffold in the lumen of the complex, formed by two copies of MZT1, actin and the N-termini of GCP6 and one copy of GCP3. This ‘luminal bridge' follows the inner surface of the spiral on the level of the GRIP1 domains from spokes 2 to 11 [12]. Further proteins that have been established to cooperate and co-purify with the γ-TuRC in a defined stoichiometry, such as NEDD1 and NME7 [13,14], could not be detected in the cryo-EM structures, probably because these proteins are flexibly associated [8].

γ-tubulin complexes have been proposed to promote *de novo* formation of microtubules by acting as structural templates [15]. The arrangement of the first eight γ-tubulin molecules of the γ-TuRC spiral indeed almost recapitulates the arrangement of *α*β-tubulin heterodimers in microtubules. However, from spoke 9 onwards, a clear deviation of γ-tubulin arrangement from microtubule symmetry could be observed, possibly ‘intentionally' limiting the ability of the γ-TuRC to function as a microtubule nucleation template [8–10]. Regulation of γ-TuRC activity thus might be realized by a ‘simple' conformational change of the γ-TuRC diameter. However, how the γ-TuRC is activated in detail remains unclear.

While reconstitution of the simpler heterotetrameric γ-tubulin small complex (γ-TuSC) from lower eukaryotes was achieved [15–18], our structural and basic functional understanding of the vertebrate γ-TuRC to date exclusively relies on purified native complexes [8–10]. The lack of a recombinant reconstitution system for the γ-TuRC particularly hampers experiments addressing the mechanistic role of γ-TuRC structural determinants in complex assembly, activation and microtubule nucleation. Based on the structural census of γ-TuRC core components obtained from recent cryo-EM studies, we have developed a reconstitution system for the human γ-TuRC that is based on the co-expression of all γ-TuRC subunits in insect cells and a gentle one-step purification strategy. We show that the recombinant γ-TuRC has the same structural organization as the native purified γ-TuRC and is active in microtubule nucleation and microtubule minus end capping. This system is a major step towards closing the gap between structural and functional/mechanistic understanding of γ-TuRC-mediated microtubule nucleation.

## 2. Material and methods

### 2.1. Molecular cloning

*GCP2*, *GCP3* and *GCP4* cDNA were a gift of Andreas Merdes (Toulouse), *GCP5* cDNA was from the DKFZ, Heidelberg collection, *GCP6* from Ingrid Hoffmann (DKFZ, Heidelberg) [19], human γ-tubulin (*TUBG1*) from Tim Stearns (Stanford), *MZT1* from Jens Lüders (Barcelona) and the beta-actin (*ACTB*) gene was obtained from AddGene. Cloning was based on the MultiBac (Geneva Biotech) expression system [20], with adapted vectors, where all plasmids contain the polH expression cassette as described in Eustermann *et al*. [21]. Each of the human genes, *TUBG1*, *GCP2*, *GCP3*, *GCP4*, *2xFLAG*-*GCP5*, *GCP6*, *ACTB* and *MZT1* were cloned via InFusion cloning (Takara) using Q5 DNA polymerase (NEB) with standardized primer design for a direct insertion following the polH promoter (figure 1*b*; electronic supplementary material, table S1 and S2). Two gene cassettes were combined via infusion cloning using the primers listed in (electronic supplementary material, table S2). GCP5 was tagged with 2xFLAG-TEV side in two subsequent PCR reactions. The generated modules were combined via subsequent Cre-recombination using Cre-recombinase (NEB) following the MultiBac manual (Geneva Biotech, version 5.1). Constructs for Cre-recombination were pACEBac1 (*2xFLAG-GCP5* or *MZT1*), pIDC (*GCP6* or *GCP2*, *GCP3*) pIDK (*TUBG1*, *GCP4*) and pIDS (*ACTB*). Genes of the different modules were verified via PCR amplification and sequencing.

### 2.2. Protein expression and purification

Bacmid production was performed as described in the MultiBac manual. Bacmids were used for virus production in Sf21 insect cells with cellfectin II reagent (Thermo Fisher Scientific). Viruses were amplified in 30 ml (1 × 10^6^ cells ml^−1^) and diluted 1 : 100 in 100–400 ml (1 × 10^6^ cells ml^−1^) expression culture in Sf21 or Hi5 cells using Sf-900 III medium (Thermo Fisher Scientific). Insect cells were infected with constructs 1 and 2 (figure 1*b*). In some experiments, human γ-tubulin with a C-terminal Myc-His_6_ tag was co-expressed using a third virus [7,22] (figure 3*a*). Infected cells were kept at 27°C for 60 h, harvested via centrifugation (800*g* for 5 min), flash frozen in liquid N_2_ and stored at −80°C until protein purification.

For protein purification, cells were resuspended in cold lysis buffer (50 mM HEPES, 150 mM KCl, 5 mM MgCl_2_, 1 mM EGTA, 1 mM DTT, 0.1 mM GTP, pH 7.4 + 0.02% (v/v) Brij-35, and 250 units Benzonase (Sigma Aldrich), 1 complete EDTA-free protease inhibitor tablet (Roche) per 15 ml lysis buffer) and kept on ice. Resuspended cells were sonicated (3 × 1 min with 0.6 amplitude, Hielscher UP50H) and centrifuged at 20 000*g* for 60 min at 4°C. Anti-FLAG M2 Affinity Gel (Sigma Aldrich) was equilibrated in lysis buffer and incubated with the centrifuged and filtered lysate (Whatman sterile filters 0.45 µm pore size) for 90 min rotating at 4°C. Afterwards beads were washed once with lysis buffer and twice with wash buffer (50 mM HEPES, 150 mM KCl, 5 mM MgCl_2_, 1 mM EGTA, 0.5 mM DTT, 0.1 mM GTP, pH 7.4). Elution was done with one bead volume of elution buffer (wash buffer plus 0.2 mg ml^−1^ 3xFLAG peptide; Gentaur) and 20 min incubation at 4°C. Elution was repeated once, and samples were used for subsequent experiments or flash frozen in liquid N_2_ and stored at −80°C. For samples which were purified for size exclusion chromatography (SEC), all buffers contained 5% (w/v) glycerol. Native γ-TuRC purified from *X. laevis* egg extract was performed as described previously [8]. SDS gels were run together with Page Ruler Plus (Thermo Fisher Scientific) on precast gradient gels (4–20% BIO-RAD) and stained with Coomassie Brilliant Blue G250 (Sigma Aldrich) or used for immunoblot analysis of the γ-TuRC components as described previously [8] with the antibodies listed in electronic supplementary material, table S3 and figures S2 and S3.

### 2.3. Fluorescence-based tubulin polymerization assay

MT nucleation was performed using the fluorescence-based Tubulin Polymerization assay kit (Cytoskeleton, Denver Com cat. no. BK011P) following the manufacturer's instructions. Briefly, 5 µl or 10 µl of the samples (buffer control (elution buffer/SEC buffer), human recombinant γ-TuRC, human recombinant γ-TuRC after size exclusion chromatography (SEC), *X. laevis* γ-TuRC or 3 µM paclitaxel (final concentration)) was pipetted into a 96-well microtiter plate, prewarmed and mixed with 45 µl of tubulin buffer (2 mg ml^−1^ porcine tubulin in 80 mM PIPES pH 6.9, 2 mM MgCl_2_, 0.5 mM EGTA, 1 mM GTP, 15% (w/v) glycerol). The concentration of human recombinant γ-TuRC after SEC and native *X. laevis* γ-TuRC were normalized to γ-tubulin signal in immunoblot analysis and diluted in SEC buffer prior to nucleation measurements accordingly. The polymerization reaction was triggered by transferring the plate to the 37°C chamber of the plate reader. Fluorescence was measured at 37°C using the plate reader for 60 min at 1 min intervals (CLARIOstar, BMG Labtech, excitation, F: 360-10, emission, F: 450-10). Data were processed using PRISM software (GraphPad v. 8).

### 2.4. *In vitro* microtubule nucleation assay for electron microscopy analysis

Thirty micromolar of porcine brain tubulin containing 4% Cy3-labelled tubulin in 1x BRB80 buffer (80 mM PIPES/KOH pH 6.8, 1 mM MgCl_2_, 1 mM EGTA and 12.5% (w/v) glycerol) was centrifuged (5 min, 352 860*g*, 4°C) with S100-AT3 rotor (Thermo Fisher Scientific). Supernatant was mixed with γ-TuRC premix (1 : 20 dilution of γ-TuRC, 1 mM GTP in 1x BRB80, 12.5% (w/v) glycerol) in ratio 1 : 1. Samples were incubated for 15–30 min on ice and transferred to a 37°C water bath for 3 min for MT polymerization. After MT polymerization samples were immediately cross-linked with 1% glutaraldehyde and used for negative stain EM.

### 2.5. Size exclusion chromatography

SEC was performed either with ‘Superose 6 (10/300) GL' or ‘Superose 6 Increase (10/300) GL' (GE Healthcare) equilibrated with SEC buffer (50 mM HEPES, 150 mM KCl, 5 mM MgCl_2_, 1 mM EGTA, 0.5 mM DTT, 0.1 mM GTP, 5% glycerol (v/w), pH 7.4). Only for runs with the ‘Superose 6 (10/300) GL' column, FLAG elutions were concentrated (Amicon, 30 K) before injection into the column. Runs with size markers thyroglobulin (669 kDa and aldolase 158 kDa) were performed separately. Peak fractions were aliquoted and flash frozen in liquid N_2_ for storage at −80°C. Samples of peak fractions were also used for ethanol precipitation (90% EtOH, −20°C, overnight) for subsequent immunoblot analysis.

### 2.6. Negative stain electron microscopy grid preparation and data collection

Five microliters of sample was applied on glow-discharged copper-palladium 400 EM mesh grids covered with an approximately 10 nm-thick continuous carbon layer. After 30 s incubation at 23°C, grids were blotted with a Whatman filter paper 50 (cat no. 1450-070) and washed on 3 drops of water. Sample on grids was stained with 3% uranyl acetate in water. Grids with nucleated microtubules were imaged at a Jeol JE-1400 (Jeol Ltd., Tokyo, Japan) operating at 80 kV equipped with a 4 k × 4 k digital camera (F416, TVIPS, Gauting, Germany). Micrographs were adjusted in brightness and contrast using ImageJ. Negative stain EM data for two- and three-dimensional class averaging of native *X. laevis* γ-TuRC [8] and recombinant human γ-TuRC were acquired on a Talos L120C TEM equipped with 4 k × 4 K Ceta CMOS camera (Thermo Fisher Scientific). To compensate for the preferred orientation of γ-TuRC particles on the continuous carbon grids, data were partially acquired at a stage tilting angle of 20 degrees. Data were acquired using EPU (Thermo Fischer Scientific) at a nominal defocus of −3 µm and an object pixel size of 0.4125 nm.

### 2.7. Negative stain electron microscopy data processing

In total, we acquired approximately 1600 micrographs of the recombinant human γ-TuRC (957 micrographs untilted, 640 micrographs at 20° stage tilt), approximately 1100 micrographs of the native *X. laevis* γ-TuRC (500 micrographs not tilted, 600 micrographs at 20° stage tilt) and 500 micrographs of recombinant human γ-TuRC after SEC without tilt. Image processing for all datasets was performed in Relion 3.1 Beta [23]. The contrast transfer function (CTF) of micrographs was estimated using gCTF [24]. For all datasets, approximately 1000 particles were selected manually and extracted at a pixel size of 0.825 nm with a box size of 64 pixels. Extracted particles were subjected to two-dimensional classification with 50 classes, a translational search range of 20 pixels at 2 pixels increment and a mask diameter of 450 Å. Two-dimensional classes unambiguously representing γ-TuRCs were selected and used as references for automated particle picking on all micrographs. The overall number of picked particles was 374 389 for the recombinant human γ-TuRC, 192 845 for the native *X. laevis* γ-TuRC and 38 139 for the recombinant human γ-TuRC after SEC. Extracted particles from each dataset were subjected to several rounds of to two-dimensional classification with 100–200 classes, a translational search range of 20 pixels at 2 pixels increment and 450 Å mask size. For three-dimensional classification particles included in true-positive classes were merged, yielding 12 402 particles of the recombinant human γ-TuRC and 12 271 particles of the native *X. laevis* γ-TuRC. Next, particles were aligned in three-dimensional using three-dimensional classification with 1 class and a translation search range of 20 pixels at 2 pixels increment. As a reference, we used a strongly low-pass filtered cryo-EM density of the γ-TuRC [8], from which we removed the GRIP2 domains and γ-tubulins of spokes 5 and 6. The aligned particles were subjected to the second round of three-dimensional classification into 6 classes without image alignment, focused on the spokes that were artificially removed from the reference in the first classification run. This allowed us to separate correctly aligned true-positive particles (with density for spokes 5 and 6) from false positive or incorrectly aligned particles (without density for spokes 5 and 6). The retained particles (6253 particles for the recombinant human γ-TuRC; 6827 particles for the *X. laevis* γ-TuRC) were submitted to another round of three-dimensional classification into 3 classes with image alignment and the best classes were selected for further structural analysis (2064 particles of the recombinant human γ-TuRC; 2490 particles of the native *X. laevis* γ-TuRC).

## 3. Results

### 3.1. A recombinant system for the expression of gamma-ring complex proteins, γ-tubulin, actin and mitotic spindle organizing protein 1 in insect cells

At least eight different proteins assemble in defined stoichiometry to form the γ-TuRC [25]. In order to achieve expression of all subunits, we decided to subclone human γ-TuRC component genes into a modified MultiBac expression system [20]. Instead of using the multiple cloning site, we followed an InFusion cloning strategy with vectors carrying the polyhedrin (polH) expression cassette (figure 1*b*) [21]. Either two gene cassettes were combined into one vector, or single gene vectors were used for subsequent Cre-recombination. This strategy allows for easy modification and shuffling of the expressed genes (figure 1*b*). As GCPs are large proteins, genes of interest were arranged on two final plasmids to avoid construction of one oversized plasmid. The two plasmids were used for virus production: construct 1 contained *2xFLAG-GCP5*, *GCP4*, *GCP6*, *TUBG1 (*coding for γ-tubulin) and *ACTB* (coding for β-actin), which were suggested to form an initial stable core complex during the assembly of γ-TuRC [8,26]; construct 2 contained *MZT1*, *GCP2* and *GCP3*, the ‘minimal set' of additional subunits required to reconstitute the 14-spoke γ-TuRC including the luminal bridge as observed in cryo-EM studies [8–10,12].

For protein expression, insect cells were infected with equal amounts of viruses and harvested after 60 h. One of the GCP subunits, GCP5, was tagged with 2xFLAG at its N-terminus for gentle single-step affinity purification of the complex from a relatively small cell culture volume (see methods) (figure 1*c*). Negative stain EM showed regular ring-shaped structures on the micrographs (figure 1*d,* boxed particles), indicating complex formation. This is supported by the detection of other γ-TuRC components co-purifying with *2xFLAG-GCP5* (figure 1*e*). As observed in other studies before [8,10], the signal intensity of the γ-tubulin band in Coomassie Blue-stained SDS gels does not reflect its high stoichiometry in the complex. The identity of several γ-TuRC components was further confirmed by immunoblotting (figure 1*f*). In conclusion, we have established an insect cell-based recombinant expression system for reconstitution of the human γ-TuRC, which allows complex purification in a one-step protocol.

### 3.2. Recombinant and native γ-tubulin ring complex are structurally indistinguishable by negative staining electron microscopy

**Figure 2.**
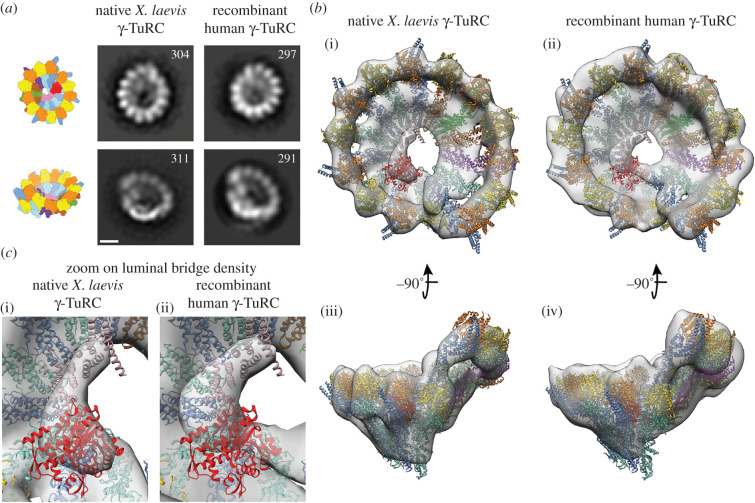
Negative stain EM analysis of recombinant human and native *X. laevis* γ-TuRCs. (*a*) Comparison of representative two-dimensional classes from the native *X. laevis* (left) and recombinant human γ-TuRC (right) in two different views, as indicated in the cartoons. Number of particles in each class is given. Scale bar: 10 nm. (*b*) Three-dimensional EM densities of (i,iii) the native *X. laevis* (2490 particles) and (ii,iv) recombinant human γ-TuRC (2064 particles) in two different views. The atomic model of the human γ-TuRC (PDB-6V5 V) [9] was docked as a rigid body and superposed to the EM densities. Colouring as in figure 1*a*. (*c*) Zooms focused on the luminal bridge density of the native *X. laevis* (i) and recombinant human γ-TuRC (ii). The same colouring as in figure 1*a*.

To verify the integrity and correct assembly of the reconstituted γ-TuRC, we performed negative stain EM. The structures of recombinant human and native *X. laevis* γ-TuRCs were compared by two-dimensional (figure 2*a*) and three-dimensional class averaging (figure 2*b*). The EM densities recapitulate the structure of the γ-TuRC as previously determined using cryo-EM, including the 14-spoke overall organization of the complex and the luminal bridge on the inner surface of the γ-TuRC (figure 2*b*). Visibility of the ordered ‘luminal bridge' structure (figure 2*c*) indicates that MZT1 and actin were correctly integrated into the complex. Importantly, the native and recombinant γ-TuRCs were structurally indistinguishable in the resolution range accessible with negative stain EM, as judged by the fit of atomic models into the two densities (figure 2*b*).

### 3.3. Size exclusion chromatography enables separation of recombinant γ-tubulin ring complex from sub-complexes

**Figure 3.**
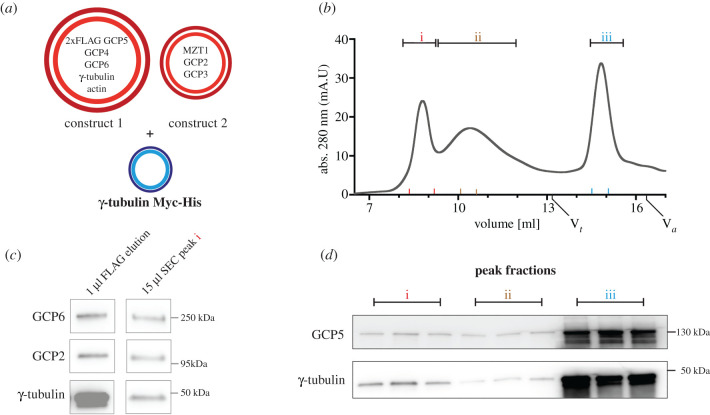
Size exclusion chromatography of recombinant human γ-TuRC. (*a*) For SEC experiments, construct 1 (2x*FLAG-GCP5*, *GCP6*, *GCP4*, *TUBG1* and *ACTB*) and construct 2 (*MZT1*, *GCP2* and *GCP3*) were co-expressed with C-terminal Myc-His tagged γ-tubulin and purified via FLAG purification. (*b*) Chromatogram of a SEC run with ‘Superose 6 Increase (10/300) GL' column. Peak fractions (i (γ-TuRC peak), ii, iii) were analysed via immunoblotting (*c*,*d*). Red, brown and blue markers on x-axes (*b*) indicate borders of analysed fractions (i, ii, iii) in (*d*). Size markers thyroglobulin 669 kDa (V_t_) and aldolase 158 kDa (V_a_) are indicated. (*c*) FLAG elution left and ‘γ-TuRC peak' fraction (i, *b*) were probed by immunoblotting against the indicated antibodies. (*d*) Immunoblot analysis of ethanol precipitated peak fractions (i, ii, iii) from SEC experiment shown in (*b*). Equal amount of fraction volumes was loaded. See electronic supplementary material, figure S2 for uncropped images.

To further optimize γ-TuRC reconstitution, we repeated the recombinant expression of γ-TuRC components with additional co-expression of C-terminal tagged (Myc-His_6_) human γ-tubulin [7] (figure 3*a*) that appeared substoichiometric in figure 1. In addition, we included size exclusion chromatography (SEC) as an analysis step to judge the ratio of fully assembled γ-TuRC relative to sub-complexes (figure 3*b*; electronic supplementary material, figure S1). The size exclusion chromatogram of the recombinant γ-TuRC (figure 3*b*) showed an early high molecular weight peak (figure 3*b*,i), which contained γ-TuRC components, as judged by immunoblotting (figure 3*c,d*)*.* Adjacent lower molecular weight peak fractions (figure 3*d*,ii) also contained γ-TuRC components but due to their running behaviour probably correspond to γ-TuRC sub-complexes (electronic supplementary material, figure S1*a*,*b*). The presence of intact γ-TuRC in early SEC elution fractions was further confirmed by negative stain EM from another γ-TuRC preparation that after concentration contained fewer medium to low molecular weight γ-tubulin sub-complexes (electronic supplementary material, figure S1*c*–*e*). SEC analysis thus confirms the integrity of the recombinant γ-TuRC and additionally suggests the presence of stable γ-TuRC assembly intermediates that will be central for dissecting the assembly pathway of this megadalton complex.

### 3.4. The recombinant γ-tubulin ring complex has microtubule nucleation activity and caps the minus ends of microtubules

**Figure 4.**
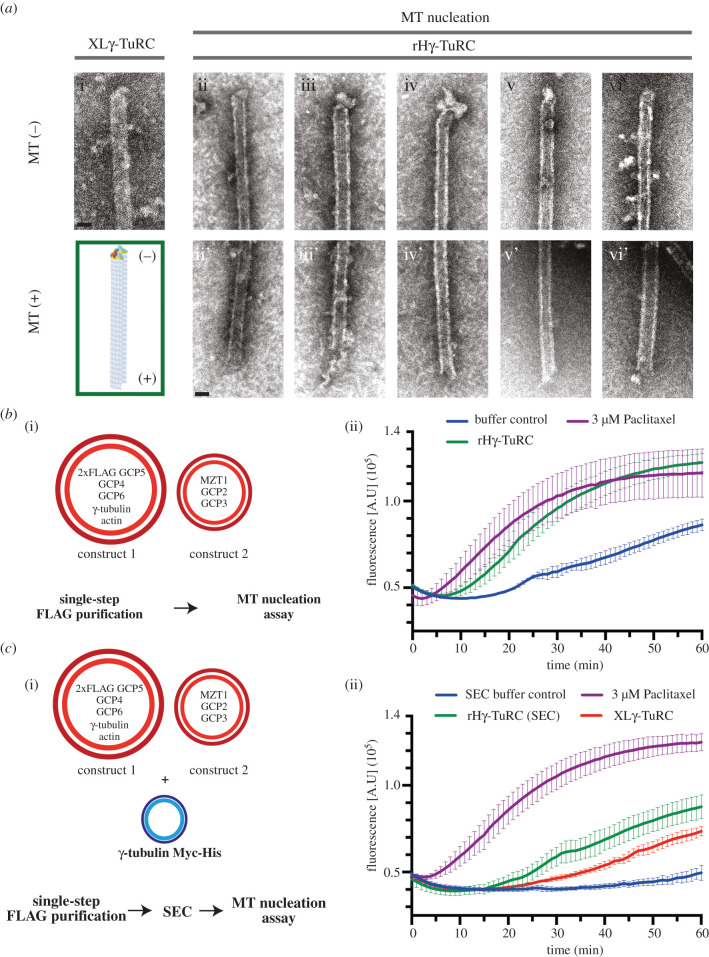
The recombinant human γ-TuRC has microtubule nucleation and minus end capping activity. (*a*) Representative negative stain EM images from microtubules nucleated by native *X. laevis* γ-TuRC; (i; XLγ-TuRC) or the recombinant human γ-TuRC (ii-vi, rHγ-TuRC). For microtubules nucleated by the recombinant human γ-TuRC, the capped microtubule minus ends (ii–vi; MT-) and the flared or sheet microtubule plus ends (ii'–vi'; +MT) are shown. A schematic representation of a γ-TuRC capped microtubule is shown in the green box. Scale bars, 25 nm. (*b,c*) Tubulin polymerization assay where the increase in fluorescence intensity over time represents *α*β-tubulin polymerization into microtubules. Shown are error bars for the standard deviation of the mean of three (*b*) and four (*c*) technical replicates. (*b*) The recombinant human γ-TuRC (construct 1 and construct 2 in figure 1, rHγ-TuRC) was analysed together with elution buffer without recombinant γ-TuRC as negative control, and 3 µM Paclitaxel as positive control. (*c*) Recombinant human γ-TuRC (construct 1, construct 2 and C-terminal Myc-His6 tagged γ-tubulin) was purified by FLAG affinity purification and subsequent SEC (figure 3*b* i). The γ-tubulin content of ‘γ-TuRC peak’ fraction (rHγ-TuRC SEC) and native *X. laevis* γ-TuRC (XLγ-TuRC) was determined by immunoblotting. Samples were diluted in SEC buffer to equal γ-tubulin concentrations and were then used for the microtubule nucleation assay with SEC buffer as a negative control and 3 µM Paclitaxel as positive control.

Having confirmed the structural integrity of the recombinant γ-TuRC, we next aimed at characterizing its functional properties. The γ-TuRC initiates assembly of microtubules by forming a structural template for the recruitment of the initial *α*β-tubulin subunits and then remains stably associated with the minus end of the growing microtubule [10,27–29]. Consistently, when analysing microtubules nucleated by the recombinant γ-TuRC via negative stain EM, we frequently observed a cap-like structure representing the γ-TuRC on one of the microtubule ends (figure 4*a*). The opposite end of these microtubules always had a frayed or open appearance as typically observed for the microtubule plus end [30,31], suggesting that the γ-TuRC is localized on the microtubule minus end (figure 4*a*). Furthermore, to determine microtubule nucleation activity of the recombinant γ-TuRC, we used a fluorescence-based microtubule nucleation assay (figure 4*b,c*). Upon the addition of the recombinant γ-TuRC after FLAG purification, microtubule assembly was clearly increased compared to elution buffer without γ-TuRC, which served as a negative control (figure 4*b*). Next, we analysed the nucleation activity of the recombinant γ-TuRC after FLAG elution and SEC (figure 4*c*) and—as an additional control—compared it to the nucleation activity of affinity-purified *X. laevis* γ-TuRC [8], normalized to the γ-tubulin content of the two preparations. We observed that the microtubule nucleation activity of the recombinant γ-TuRC was comparable to the affinity-purified *X. laevis* γ-TuRC. Collectively, these data confirm that the recombinantly expressed and purified γ-TuRC has microtubule nucleation activity.

## 4. Discussion

Here, we report a strategy for recombinant expression, reconstitution and purification of the human γ-TuRC from small cell culture volumes of insect cells. This system enables fast and efficient screening of structural and functional properties of genetically modified γ-TuRC variants in follow-up experiments. The success of this strategy, which is based on FLAG-GCP5 affinity purification, indicates that the N-terminus of GCP5, which has not been completely traced in the recent cryo-EM reconstructions, is accessible for antibody binding and likely not as deeply embedded in the γ-TuRC as the GCP6 N-terminus [12]. This FLAG-tagged γ-TuRC variant thus can likely be immobilized in a defined orientation for single-molecule TIRF microtubule nucleation assays [10,29], which require the γ-tubulin face of the γ-TuRC to be accessible (figure 1*c*). Such an approach will allow comparing microtubule nucleation activity of γ-TuRC species with differing subunit composition or mutations.

SEC as an additional purification step furthermore indicated that our purification strategy yields not only fully assembled recombinant γ-TuRC, but also sub-complexes that may represent intermediates in γ-TuRC assembly (figure 3; electronic supplementary material, figure S1). Moreover, our experiments show that expression of *TUBG1, GCP2* to *GCP6*, *ACTB* and *MZT1* with or without the excess of tagged *TUBG1* was sufficient for reconstitution of the γ-TuRC. Thus, the γ-TuRC targeting factor NEDD1 and the nucleation regulator NME7 seem to be not required for complex assembly, consistent with previous data [13,14]. Similarly, since the human γ-TuRC was reconstituted in the absence of *MZT2*, which is not encoded in insect cells [32], we can conclude that MZT2 is not essential for structural integrity and microtubule nucleation activity of the γ-TuRC. This is consistent with the recently proposed function for MZT2 as an adaptor of the γ-TuRC for MTOC binding [33]. By contrast, the two molecules of MZT1 integrated into the luminal bridge scaffold clearly fulfil a structural role [12] and are probably required for the formation of a stable complex. Such a function is consistent with the observation that *MZT1* depletion affects the integrity of the γ-TuRC [17].

Our recombinant expression system now places us in a position to address central questions regarding γ-TuRC assembly, activation and γ-TuRC-associated microtubule nucleation from a mechanistic perspective. A prime candidate for detailed mutational analysis is the long insertion domain between the GRIP1 and GRIP2 domains of GCP6, which has been identified to interact specifically with the N-termini of GCP2 and GCP5, and therefore to potentially assist in the assembly of the γ-TuRC [8]. Our system will allow us to analyse the function and structure of γ-TuRC variants assembled in the presence of mutated versions of this GCP6 insertion domain. In a similar manner, we will be able to address the structural and functional role of actin in the γ-TuRC, which has remained elusive so far. Collectively, our recombinant γ-TuRC expression system opens up many possibilities for structure-function analyses of this microtubule nucleator and its cooperation with additional factors, such as XMAP215 and the augmin complex [34,35].

## Data accessibility

This article has additional data in the figures and tables of the electronic supplementary material.
